# Pharmacy students’ interprofessional experience and performance in advanced pharmacy practice experience rotations amid COVID-19 pandemic

**DOI:** 10.3389/fmed.2024.1394652

**Published:** 2024-05-21

**Authors:** Marwan Alrasheed, Abdulrahman Alsuhibani, Wadha Alotaibi, Musaab Gari, Amin Alashgar, Michael Doherty, Jill Boone, Thamer A. Almangour, Ana Hincapie

**Affiliations:** ^1^Department of Clinical Pharmacy, College of Pharmacy, King Saud University, Riyadh, Saudi Arabia; ^2^James L. Winkle College of Pharmacy, University of Cincinnati Academic Health Center, Cincinnati, OH, United States

**Keywords:** APPE, training and development, pharmacy, COVID-19, education

## Abstract

**Introduction:**

Interprofessional education (IPE) is essential in pharmacy training, providing students with vital collaborative skills for real-world healthcare. Advanced Pharmacy Practice Experience (APPE) is integral to IPE, allowing students to apply their knowledge in diverse healthcare settings. The COVID-19 pandemic has disrupted healthcare education and raised concerns about its impact on IPE during APPE rotations. Our study investigates the pandemic’s influence on pharmacy students’ interprofessional interactions and APPE performance.

**Objective:**

To assess the interprofessional experiences of fourth-year pharmacy students before and during the COVID-19 pandemic in the context of APPE.

**Methods:**

This retrospective observational study examined the experiences of P4 pharmacy students in the United States during APPEs before and during the pandemic. We employed electronic surveys with 21 questions to gauge interactions and interprofessional team effectiveness, employing Likert scale response options. We compared responses between the 2019–2020 and 2020–2021 APPE rotations using statistical tests.

**Results:**

Our study encompassed 83 and 86 students for the 2019–2020 and 2020–2021 APPE rotations, respectively, achieving a 100% response rate. Amid the pandemic, written communications between pharmacy students and healthcare providers in general medicine rotations increased, while in-person engagement decreased. Pre-COVID, students reported higher colleague referrals and greater interprofessional utilization during ambulatory care rotations.

**Conclusion:**

COVID-19 shifted interactions from in-person to written communication between pharmacy students and healthcare providers. Students reported decreased satisfaction with their interprofessional experiences. This research offers insights into the changing landscape of pharmacy education, helping students prepare for evolving challenges in healthcare delivery and education.

## Introduction

Interprofessional education (IPE) stands as a pillar of pharmacy education, equipping students with the essential skills to effectively collaborate within healthcare teams (1). The advanced pharmacy practice experience (APPE) provides students with a valuable opportunity to put their knowledge and skills into practice in real healthcare settings, fostering the development of their interprofessional abilities. However, the onset of the COVID-19 pandemic wrought significant changes in healthcare education and delivery, including pharmacy education, giving rise to concerns about the impact on students’ IPE during APPE rotations (2). The pandemic has ushered in a transformation in healthcare delivery, marked by the increased use of telemedicine and stringent adherence to social distancing measures (3). These shifts have posed challenges for pharmacy students in their learning and collaborative endeavors with other healthcare professionals. For instance, students now have fewer chances to observe professionals in their work, accompany them on hospital rounds, or engage in interactive discussions and feedback sessions during telemedicine encounters (4, 5). Moreover, the pandemic has added stress and heightened workloads for all healthcare practitioners, including pharmacy students, making it arduous to devote attention to their interprofessional growth.

To address these challenges, pharmacy schools have undertaken measures to mitigate the adverse effects of the pandemic on students’ IPE during APPE rotations. For instance, schools are devising innovative IPE activities tailored to the constraints of social distancing and telemedicine, such as online simulations or collaborative case studies that require students to work together remotely (4). Additionally, schools are offering enhanced support to help students develop their interprofessional skills through workshops focusing on communication, teamwork, and conflict resolution (6). Furthermore, pharmacy schools are forging partnerships with other healthcare education institutions to create opportunities for interprofessional collaboration. These collaborations encompass initiatives like joint IPE programs, interprofessional events, and shared rotations or clinical placements, fostering a rich environment for students to engage in meaningful interprofessional experiences (7).

This study seeks to study the impact of the COVID-19 pandemic on pharmacy students’ interprofessional experiences and performance during their APPE rotations. The primary objectives of this research encompass a comprehensive examination of the interactions and interprofessional encounters of fourth-year pharmacy students with healthcare professionals, both before and during the COVID-19 pandemic, within the context of APPE. Through this inquiry, we aim to glean valuable insights that will inform strategies and interventions to bolster the resilience and adaptability of pharmacy education in the face of future uncertainties or disruptions.

## Methods

This was a descriptive retrospective observational study assessing the fourth-year pharmacy students’ experiences during APPEs before (2019–2020) and during the COVID-19 (2020–2021) pandemic, these two groups were independent of one another. The census approach was utilized to systematically gather comprehensive data on APPE experiences, ensuring a thorough analysis of all pharmacy interns. The study was conducted at a large public university in the United States. An Electronic survey composed of 21 items was distributed to students at the end of each rotation to capture their experiences with the rotation. The survey was adopted from the Modified Index of Interdisciplinary Collaboration (MIIC) (8). The MIIC has been shown to be a reliable and valid measure of interdisciplinary collaboration in a variety of settings, including healthcare, education, and social services. It has been used to assess the impact of interventions designed to improve interdisciplinary collaboration, and to identify areas where teams can improve their collaboration practices. The survey was pilot tested with a small group of students to ensure that the items were clear and easy to understand. The survey elicited experiences in two dimensions: students’ interactions and the teams’ effectiveness. Students were asked at the end of each rotation to rate the frequency and quality of their interactions with other healthcare professionals, such as physicians, nurses, and pharmacists. Students were also asked to rate the effectiveness of their teams in terms of communication, collaboration, and decision-making (Supplementary material).

## Statistical analysis

We compared students’ answers between APPE rotations before and during COVID-19 using the chi-square and Wilcoxon rank-sum tests. These statistical tests were used to compare the distribution of responses between the two groups of students. The chi-square test was used to compare categorical variables, such as the frequency of interactions with other healthcare professionals. The Wilcoxon rank-sum test was used to compare continuous variables, such as the rating of team effectiveness. To measure the internal consistency and reliability of a scale used in this study, Cronbach’s Alpha test has been used. Alpha level at 0.05 was used for all statistical tests to maintain a standard balance between type I error rate and statistical power, ensuring conservative significance testing. Also, two-tailed test was employed to detect any significant differences in both directions, thus ensuring we did not miss effects due to predetermined expectations. A *post hoc* analysis using G*Power version 3.1.9.6 was conducted to ascertain the minimum sample size required to effectively test the study hypothesis. This analysis indicated that to achieve 80% power for detecting a medium effect size, with a significance level of *α* = 0.05, a sample size from 120 to 134 is necessary when utilizing Wilcoxon and chi-square tests. The actual sample sizes of *N* = 83 and *N* = 86 (total *N* = 169) for the respective groups comfortably exceed this threshold, ensuring robust hypothesis testing (9, 10).

## Results

**Figure 1:**
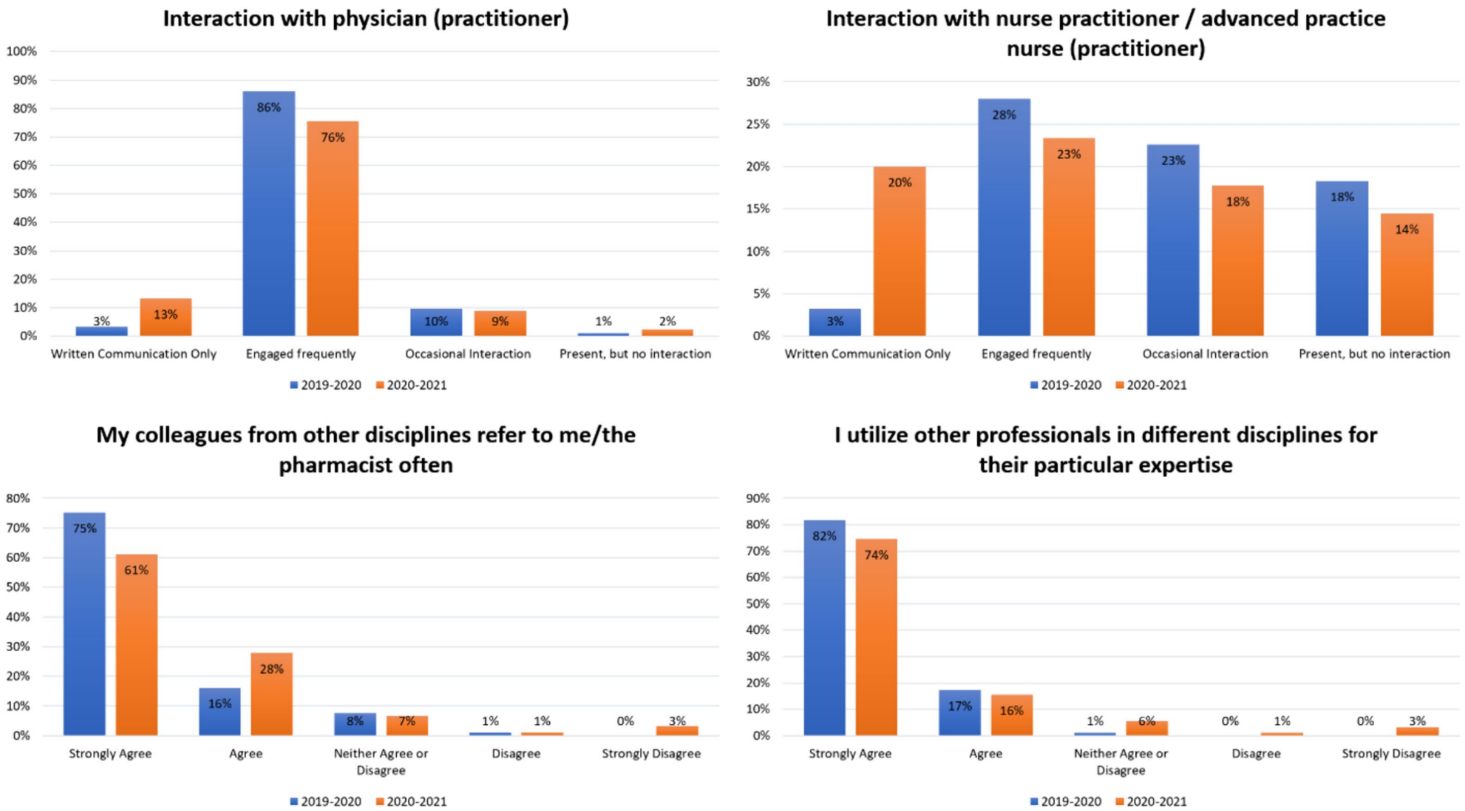
Students’ interactions and teams’ effectiveness in the general medicine rotation in two consecutive years.

**Figure 2:**
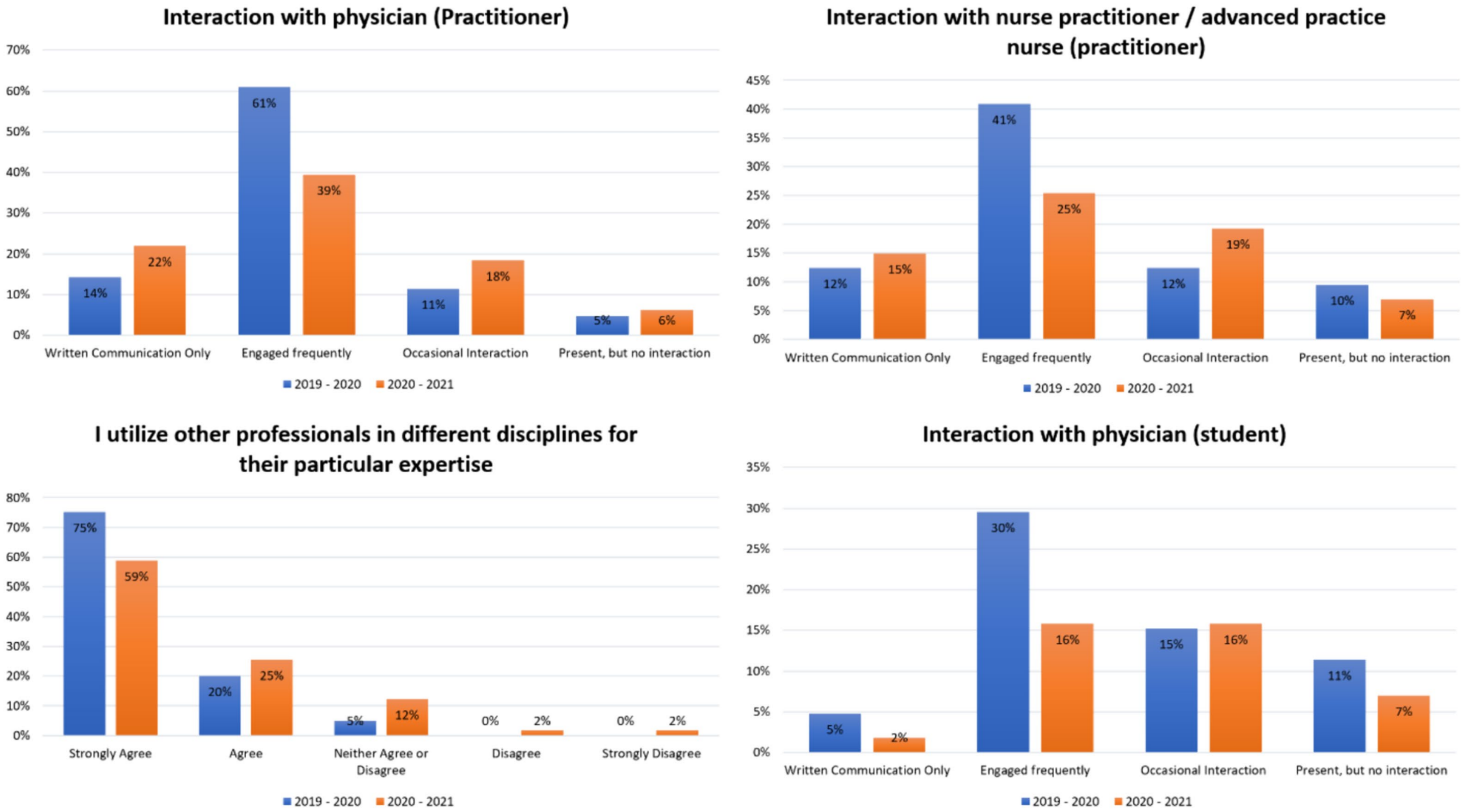
Students’ interactions and teams’ effectiveness in the ambulatory care at hospital rotation in two consecutive years.

A total of 83 and 86 students participated in the APPE rotations in 2019–2020 and 2020–2021, respectively. The response rate was 100%. Cronbach’s Alpha was 0.76 for the current cohort. During the COVID-19 pandemic, there was a significant increase in the proportion of pharmacy students who reported engaging in written communication with physicians and nurse practitioners in general medicine rotations from 3 to 13%, (*X*^2^ (1, *N* = 169) = 6.21, *p* = 0.012) and from 3 to 20% (*X*^2^ (1, *N* = 169) = 12.67, *p* < 0.01) (Figure 1). However, there was also a decrease in the proportion of students who reported engaging frequently with these healthcare professionals from 86 to 76% (*X*^2^ (1, *N* = 169) = 3.24, *p* = 0.071) and from 28 to 23%, (*X*^2^ (1, *N* = 169) = 0.51, *p* = 0.474) (Figure 1). Prior to the COVID-19 pandemic, 75% of students strongly agreed that their colleagues from other disciplines referred to them or the pharmacist often. However, during the pandemic, only 61% of students strongly agreed with this statement (*U* = 4,765, *p* < 0.05) (Figure 1). Similar results were observed in the medical-surgical and ambulatory care rotations, with more written communications and less engaging with different healthcare providers during the pandemic. In particular, in the ambulatory care rotation, fewer students during the COVID-19 pandemic strongly agreed that they utilized other professionals in different disciplines for their expertise (75% vs. 59%, *U* = 7,097, *p* < 0.05) (Figure 2). These findings suggest that the COVID-19 pandemic has shifted pharmacy students’ and healthcare providers’ interactions from engaging frequently to more written communications.

## Discussion

The findings of this study suggest that the COVID-19 pandemic has had a negative impact on pharmacy students’ interprofessional experience. This is concerning, as IPE and collaboration are essential for preparing pharmacy students to work effectively in real-world healthcare teams. The increase in written communications between pharmacy students and other healthcare providers during the pandemic may be due to a number of factors, such as social distancing requirements and the increased use of telemedicine (11, 12). However, it is important to note that written communication is not always as effective as face-to-face communication for building relationships and collaborating. Face-to-face communication allows for the richness of cues, such as facial expressions, gestures, and gaze, which are important for effective communication and collaboration (13). These social cues provide additional information and help to establish trust and understanding (14). In contrast, written communication lacks these cues and can lead to misinterpretation or misunderstanding (13). Furthermore, face-to-face communication allows for immediate feedback and clarification, which is crucial for building relationships and collaborating effectively (15). In written communication, there is often a time delay in receiving and responding to messages, which can hinder the flow of conversation and impede relationship-building (16). Research has shown that face-to-face communication is preferred over written communication for tasks that require collaboration and coordination. In a study on shift handover, participants found face-to-face communication to be the most preferred modality, as it reduced task errors significantly compared to written communication alone (17). This shift may have implications for pharmacy students’ interprofessional development and their ability to learn from and collaborate with other healthcare professionals. The decrease in pharmacy students’ engagement with other healthcare providers during the pandemic is also alarming, as engagement is essential for interprofessional learning and collaboration. It is possible that the decrease in engagement was due to factors such as the increased workload and emotional distress on healthcare professionals during the pandemic (18).

The fact that fewer pharmacy students during the pandemic strongly agreed that they utilized other professionals in different disciplines for their expertise is also concerning. This suggests that the pandemic may have made it more difficult for pharmacy students to learn from and collaborate with other healthcare professionals. A study conducted at the University of Alberta found that pharmacy students’ learning was significantly affected by the pandemic (19). The survey distributed to pharmacy students revealed the challenges they faced in adapting to remote learning and the limitations in their ability to interact and collaborate with other healthcare professionals (19). Additionally, the pandemic has highlighted the importance of health workforce preparedness and resilience. An article published in The International Journal of Health Planning and Management emphasized the need to learn from the COVID-19 pandemic in terms of health workforce development (20). The pandemic has exposed vulnerabilities in the healthcare system and underscored the significance of effective collaboration and coordination among healthcare professionals (20). Pharmacy students, as future healthcare workforce, may have encountered difficulties in developing these skills due to the limitations imposed by the pandemic. Overall, the results of this study suggest that the COVID-19 pandemic has had a negative impact on pharmacy students’ interprofessional experience. This is a significant concern, as IPE and collaboration are essential for preparing pharmacy students to work effectively in real-world healthcare teams. Pharmacy schools and healthcare organizations need to develop strategies to mitigate the negative impact of the pandemic on IPE and collaboration. Some possible strategies include developing new IPE activities that are compatible with social distancing and telemedicine, providing students with additional support to develop their interprofessional skills, such as workshops on communication, teamwork, and conflict resolution, and partnering with other healthcare education institutions to create opportunities for interprofessional collaboration. Moreover, creating a culture of interprofessional collaboration by fostering relationships with other healthcare professions and providing opportunities for students to collaborate with other students from different healthcare disciplines might help pharmacy students to become more effective members of interprofessional teams after graduation. By taking these steps, pharmacy schools and healthcare organizations can help to ensure that pharmacy students are prepared to work effectively in interprofessional teams in the post-pandemic world.

In the study, emphasis was placed on how healthcare professionals from various disciplines learn and collaborate effectively. The principles of Social Learning Theory were applied, highlighting that professionals can learn through observing and interacting within their workplace environments (21). Furthermore, the study incorporated the evolved concept of Communities of Practice, which clarifies how groups of professionals effectively share and develop knowledge and skills (22). These theoretical frameworks not only informed the research design but also aided in interpreting the findings, revealing insights into how educational strategies could be effectively translated into collaborative practices in healthcare. The findings suggest that enhancing interprofessional collaboration could lead to significant improvements in patient care outcomes.

The study on the impact of COVID-19 on pharmacy students’ interprofessional experience has several limitations. First, the study was conducted at a single institution, so the results may not be generalizable to other pharmacy schools or healthcare settings. Second, the study was retrospective, meaning that the researchers relied on students’ self-reported data, which may be subject to recall bias. Third, the study used a small sample size, which limits the power of the study to detect statistically significant differences between the two groups of students. Fourth, the study did not control for all potential confounding variables, such as students’ prior interprofessional education experiences or their personal experiences with the COVID-19 pandemic. Despite these limitations, the study provides valuable insights into the impact of the COVID-19 pandemic on pharmacy students’ interprofessional experiences. Future research should be conducted to address the limitations of this study. For example, researchers could conduct a prospective study with a larger sample size and control for potential confounding variables. Researchers could also explore the impact of the COVID-19 pandemic on pharmacy students’ interprofessional experiences in different healthcare settings and among students with different prior interprofessional education experiences. Lastly, one more limitation of this study stems from the unavailability of demographic data and other potential confounding variables, which restricts our ability to adjust the comparisons between the student groups from the 2019–2020 and 2020–2021 academic years. Consequently, the results should be interpreted with caution, recognizing that the absence of control for confounding factors could influence the outcomes reported.

In light of the unprecedented challenges posed by the COVID-19 pandemic and the impact it had on pharmacy students’ interprofessional experiences and performance during APPE rotations, it is imperative that we recognize the potential for similar public health crises in the future. Our findings underscore the need for comprehensive preparedness strategies within pharmacy education and healthcare systems. As COVID-19 has demonstrated, the ability of pharmacy students to adapt, collaborate, and provide essential services is crucial during such crises. Therefore, it is incumbent upon educational institutions and healthcare providers to learn from the experiences of our students and use these insights to develop robust strategies for future challenges. This includes enhancing remote learning capabilities, interprofessional communication and collaboration, and ensuring access to necessary resources. By taking proactive steps based on the lessons learned from this study, we can better equip future generations of pharmacy students to respond effectively and contribute to the healthcare system’s resilience in the face of unforeseen challenges.

## Conclusion

Amidst the COVID-19 pandemic, the dynamics of interactions between pharmacy students and healthcare providers underwent a transformation, transitioning from frequent face-to-face engagement to an increased reliance on written communications. Concurrently, students conveyed a diminished level of satisfaction with their interprofessional experiences throughout the pandemic period.

## Data availability statement

The datasets presented in this article are not readily available because data is available upon request. Requests to access the datasets should be directed to malrasheed1@ksu.edu.sa.

## Ethics statement

This study was approved to be conducted by the Institutional Review Board of University of Cincinnati FWA #: 00003152.

## Author contributions

MA: Conceptualization, Data curation, Formal analysis, Resources, Writing – original draft, Writing – review & editing. AbA: Investigation, Methodology, Project administration, Writing – review & editing. WA: Project administration, Resources, Supervision, Writing – review & editing. MG: Investigation, Methodology, Project administration, Writing – review & editing. AmA: Conceptualization, Formal analysis, Writing – review & editing. MD: Methodology, Writing – review & editing. JB: Methodology, Writing – review & editing. TA: Data curation, Project administration, Writing – review & editing. AH: Project administration, Software, Writing – review & editing.
