# Supplementary material for: Pharmacy students’ interprofessional experience and performance in advanced pharmacy practice experience rotations amid COVID-19 pandemic
Source: Front Med (Lausanne). 2024 May 21;11:1394652. doi: 10.3389/fmed.2024.1394652 (PMC11148434; doi:10.3389/fmed.2024.1394652)
Supplement: Supplementary file 1 [file Table_1.pdf]

College of Pharmacy APPE IPE ASSESSMENT

Please identify the professionals with whom you interacted and to what extent.

		Not applicable	Present, but no interaction	Occasional Interaction	Engaged frequently	Written Communication Only	Comment
Physician	Practitioner						
	Student						
Physician Assistant	Practitioner						
	Student						
Nurse Practitioner / Advanced Practice Nurse	Practitioner						
	Student						
Nurse (RN, LPN)	Practitioner						
	Student						
Nutrition/Dietician	Practitioner						
	Student						
Social Work/Case Manger	Practitioner						
	Student						
Physical/Occupational Therapist	Practitioner						
	Student						
Other (please describe)							

With respect to your interprofessional team as defined above with at least occasional interaction, please rate the effectiveness of your team.

	**Strongly Disagree**	**Disagree**	**Neither Agree or Disagree**	**Agree**	**Strongly Agree**	**Comments**
Our team works effectively together to improve patient care						
I utilize other professionals in different disciplines for their particular expertise.						
My colleagues from other disciplines refer to me/the pharmacist often.						
I can define those areas that are distinct in my professional role from that of professionals from other disciplines with whom I work						
The colleagues from other disciplines with whom I work seem to have a good understanding of the distinction between my role and their role(s).						
My colleagues from other disciplines are committed to working together.						
